# Facility Characteristics Associated With Intensity of Care of Nursing Home Residents With Advanced Dementia

**DOI:** 10.1093/geroni/igab046.874

**Published:** 2021-12-17

**Authors:** Meghan Hendricksen, Susan Mitchell, Ruth Lopez, Kathleen Mazor, Ellen McCarthy

**Affiliations:** 1 Hinda and Arthur Marcus Institute for Aging Research, Boston, Massachusetts, United States; 2 Hinda and Arthur Marcus Institute for Aging Research, Roslindale, Massachusetts, United States; 3 MGH Institute of Health Professions, Boston, Massachusetts, United States; 4 University of Massachusetts Medical School, Worcester, Massachusetts, United States; 5 Marcus Institute for Aging Research, Hebrew SeniorLife, Boston, Massachusetts, United States

## Abstract

Profound variations in care intensity of nursing home (NH) residents with advanced dementia exist for NHs within and across hospital referral regions (HRRs). Little is known about how these levels of influence relate. Nationwide 2016-2017 Minimum DataSet was used to categorize NHs and HRRs into 4 levels of care intensity based on hospital transfer and tube-feeding rates among residents with advanced dementia: low intensity NH in low intensity HRR; high intensity NH in low intensity HRR; low intensity NH in high intensity HRR; and high intensity NH in high intensity HRR. We used multinomial logistic regression to identify NH characteristics associated with belonging to each of 4-levels of intensity as compared to low intensity NH in low intensity HRRs (reference). We found high intensity NHs in high intensity HRRs were more likely to be in an urbanized area, not have an dementia unit, have an NP/PA on staff, have a higher proportion of residents who were male, age <65, of Black race, and had pressure ulcers, and relatively fewer days on hospice. Whereas in low intensity HRRs, higher proportion of Black residents was the only characteristic associated with being a high intensity NH. These findings suggest potentially modifiable factors within high intensity HRRs that could be targeted to reduce burdensome care, including having a dementia unit, palliative care training for NP/PAs, or increased use of hospice care. This study underscores the critical need to better understand the role race plays in the intensity of care of NH residents with dementia.

